# A Colorimetric Method for the Rapid Estimation of the Total Cannabinoid Content in Cannabis Samples

**DOI:** 10.3390/molecules28031303

**Published:** 2023-01-30

**Authors:** Neus Jornet-Martínez, Josep Biosca-Micó, Pilar Campíns-Falcó, Rosa Herráez-Hernández

**Affiliations:** MINTOTA Research Group, Departament de Química Analítica, Facultat de Química, Universitat de València, Dr. Moliner 50, 46100 Burjassot, Spain

**Keywords:** cannabis, total cannabinoids, colorimetric analysis, Fast Blue B

## Abstract

A colorimetric method for the estimation of the total content of cannabinoids in cannabis samples is proposed. The assay is based on the reaction of these compounds with the reagent Fast Blue B (FBB), which has been immobilized into polydimethylsiloxane (PDMS). The reaction and detection conditions have been established according to the results obtained for the individual cannabinoids Δ^9^-tetrahydrocannabidiol (THC), cannabidiol (CBD), and cannabinol (CBN), as well as for ethanolic extracts obtained from cannabis samples after ultrasonication. In contact with the extract and under basic conditions, the reagent diffuses from the PDMS device, producing a red-brown solution. The absorbances measured at 500 nm after only 1 min of exposure to the FBB/PDMS composites led to responses proportional to the amounts of the cannabinoids in the reaction media. Those absorbances have been then transformed in total cannabinoid content using CBD as a reference compound. The potential utility of the proposed conditions has been tested by analyzing different cannabis samples. The selectivity towards other plants and drugs has been also evaluated. The present method is proposed as a simple and rapid alternative to chromatographic methods for the estimation of the total content of cannabinoids.

## 1. Introduction

In recent years, a great interest in Cannabis sativa L. plants and derived products has been observed, not only due their widespread use for recreational purposes, but also because of their employment in the pharmaceutical, nutraceutical, food, and cosmetic industries [1,2]. Cannabinoids are the most abundant constituents of cannabis plants, with Δ^9^-tetrahydrocannabidiol (THC) being the main psychoactive compound. The most abundant non-psychoactive constituent is cannabidiol (CBD), although significant amounts of other cannabinoids such as cannabinol (CBN), cannabigerol, and the acidic forms of THC and CBD are also present [3,4]. The percentages of the different cannabinoids in plant materials depend on several factors such as the variety of plant, and the cultivation and harvesting practices. In addition, cannabinoids undergo different chemical reactions during the drying, storage, and transformation processes. Determining the content of some cannabinoids is necessary in certain applications. For example, because of its psychoactive properties, the content of THC is typically used to differentiate between drug-type plants and fiber-type plants, which in turn determines if they are suitable for legal usage. The content of CBD is increasingly used in relation with the therapeutical uses of cannabis plants as this compound offers several potential benefits due to their antioxidant, neuroprotective, analgesic, anti-epileptic, anti-inflammatory, and antimicrobial activity [2,5]. CBN has been proposed as a marker for the storage control of cannabis material [4]. 

Today, the accurate determination of the main cannabinoids in cannabis and derived samples is well established, with chromatographic techniques, both liquid chromatography (LC) and gas chromatography (GC), being the most widely used by far. These techniques have been widely applied to determine the content of major and minor cannabinoids and their distribution profile [2,5]. LC is generally preferred because this technique is better suited for the determination of acidic forms of cannabinoids [6], and it has been widely used under conventional reversed-phase conditions with both spectroscopic UV/diode array detection (DAD) [3,7,8] and mass spectroscopy (MS) or tandem LC-MS/MS detection [9]. Moreover, LC can be applied to the analysis of a variety of cannabis-based sample types such as plants, oils, creams, or candies, among many others [7]. Novel modalities/formats such as ultra-performance LC (UPLC) [8,10] or nano LC [11] are also increasingly used for the identification and determination of cannabinoids in different types of samples. On the other hand, GC allows the simultaneous determination of cannabinoids and volatile components of cannabis plants such as terpenes [12]. Methods based of thin-layer chromatography (TLC) after a chemical reaction, typically with the reagent Fast Blue B (FBB) or a derivative, have been also proposed [13,14]. More recently, different biosensors have been developed for the specific determination of cannabinoids, mainly THC, using different optical, electric, or gas sensors devices, some of them under portable formats [15]. Other techniques employed are near-infrared spectroscopy (NIR) [16] and nuclear magnetic resonance spectroscopy (NMR) [17].

Because of the great expansion of the cannabis market, the determination of the total content of cannabinoids can be of interest, for example, to evaluate the effect of cultivation conditions on the characteristics of the plants, to compare the potency of plants intended for the same use, or to assess the quality of raw materials and residues generated during industrial processes aimed at the production of cannabis derivatives [18,19]. Undoubtedly, the total content can be estimated from the amounts of the most abundant cannabinoids, typically THC, CBD, and CBN, previously obtained by LC or GC. However, a rapid and simplified non-chromatographic method for the rough estimation of the total content of cannabinoids would be desirable. 

In the present work, we have developed a colorimetric method for the estimation of the total content of cannabinoids in cannabis samples. The reagent FBB has been selected to transform the analytes into colored species. This reagent was proposed some time ago for the visual detection of the main cannabinoids after TLC separations [20]. After the chromatographic separation, the areas of the corresponding cannabinoids were scrapped off from the TLC plates separately, and the derivatives were extracted in ethanol. Finally, the extracts were used for the colorimetric determination of the respective cannabinoids. To the best of our knowledge, no other quantitative application of FBB for cannabinoids has been reported. In the present study, the FBB reagent has been used for the first time for the direct colorimetric determination of cannabinoids in extracts of cannabis sativa samples.

According to the literature, FBB reacts with THC, CBN, and CND, as well as with other minor cannabinoids such as cannabigerol by an azo coupling reaction [13]. Under alkaline conditions, the hydrogen of the OH group of cannabinoids can be lost, with the negative charge generated being stabilized by resonance. As a result, the phenolic group acts as an electron-donating group. The coupling of such a group with a diazonium compound (FBB in this case) produces azo aromatic compounds which, in general, tend to be brightly colored due to the extended conjugated systems generated. Therefore, it can be assumed that the color developed for extracts obtained for the sample is a good estimation of the total content of cannabinoids in the samples. 

In this study, and considering that solutions of FBB present a limited stability [20], we have used the reagent in a solid format immobilized into a PDMS support [21]. The employment of reagents immobilized into polymeric matrices offers some advantages over the solution reactions, mainly the possibility of simplifying the experimental protocol [22,23,24,25], and, in some cases, improved selectivity [26] and the longer stability of the reagent device compared with the solutions of the reagent [22,27]. The reaction and detection conditions have been established according to the results obtained for individual cannabinoids and extracts of cannabis plants. The potential utility of the proposed FBB/PDMS approach has been tested through the analysis of different forensic cannabis sativa samples. 

## 2. Results

### 2.1. Reaction in Solution

First, the reaction was studied in solution using conditions adapted from the literature [20]. The stability of the solutions of the reagent was tested by registering the absorbance of a solution of FBB within the 0–10-days interval. A significant variation in the spectra was already observed two days after the preparation of the solution. Consequently, in further experiments, only solutions of FBB freshly prepared were used. 

**Figure 1:**
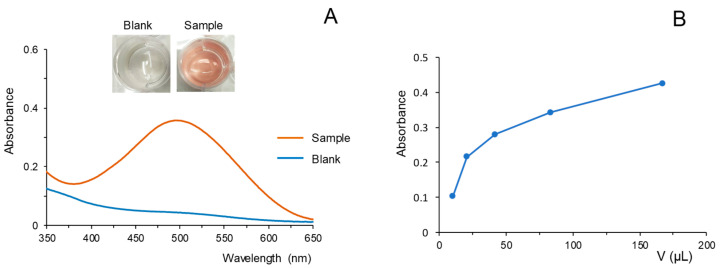
(**A**) Images and spectra obtained for a blank and an extract of cannabis using the reaction in solution. (**B**) Variation of the absorbance at 500 nm (corrected by the absorbance of a blank) as a function of the volume of sample extract per mL of reaction mixture. Reaction time, 1 min. For other experimental details, see text.

Initial assays were performed by treating cannabis samples with methanol (1 mL of solvent per 2 mg of sample) in an ultrasonic bath for 10 min. Aliquots of 200 µL of the extracts obtained were then introduced into a cuvette and mixed with 200 µL of a solution of the reagent. Next, 200 µL of a solution of 0.025 M NaOH were added (time zero for the reaction). It was observed that in the presence of sample extracts, the reaction solution turned red-brown, which is consistent with the results obtained by other authors [13]. The reaction was very fast and the absorbances of the resulting solutions remained approximately constant a few seconds after the addition of the NaOH solution, which indicated that the derivatives originated were stable, at least up to 5 min (Figure S1). However, the absorbances measured when processing directly 200 µL of the extract were excessively high. Thus, a previous dilution with water was necessary to obtain suitable values. A yellowish solution was obtained for the blank, the spectra being similar to that of the reagent in water. Constant absorbances were also registered for the blank within the tested time interval (Figure S1). As an example, in Figure 1A are depicted the spectra obtained for a blank (200 µL of water) and for 200 µL of the diluted extract (50 µL of extract and 150 µL of water) after 1 min of reaction. As observed, the spectra of the sample showed a maximum around 490–500 nm. A working wavelength of 500 nm was selected as the analytical signal for analyzing the extracts obtained from cannabis samples.

Methanol or mixtures of methanol with another organic solvent are predominantly used for the extraction of cannabinoids [4]. In another set of experiments, ethanol and methanol were tested and compared for the extraction of the cannabinoids from the cannabis plants. The results obtained demonstrated that the extraction efficiency was comparable between the two solvents (Figure S2). Therefore, considering that ethanol is less harmful, this was the solvent selected for further work. Moreover, no signal corresponding to cannabinoids was observed by treating the sample residue with another portion of ethanol (see also Figure S2). This indicates that, under the selected conditions, most part of the cannabinoids of the sample were extracted with the first fraction of ethanol. 

**Figure 2:**
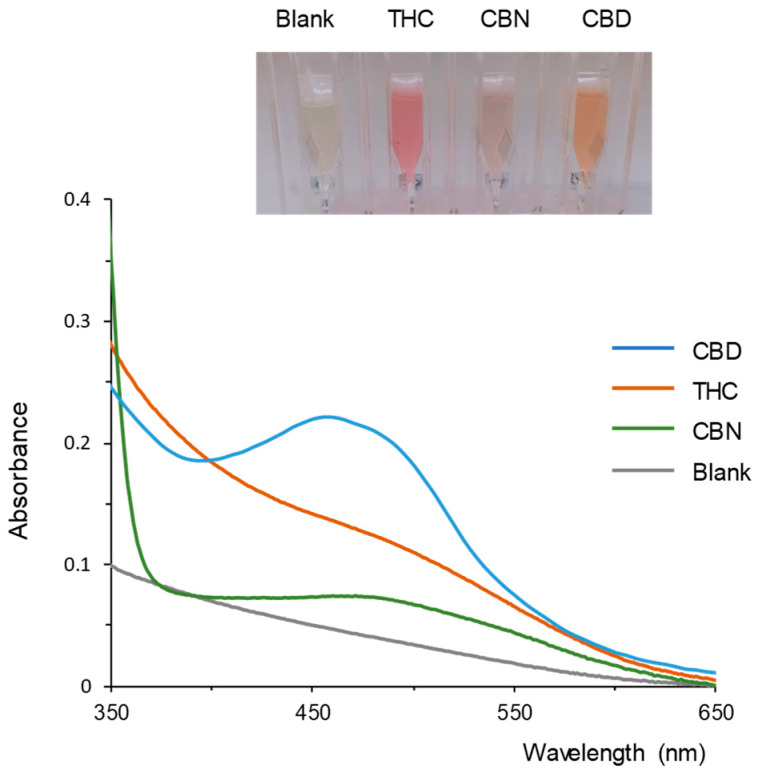
Image and spectra of the solutions obtained for a blank (water) and for the three cannabinoids tested after reaction with FBB in solution. Concentration of the analytes in the reaction solution, 25 µg/mL; reaction time, 10 min. For other experimental details, see text.

Standard solutions of individual cannabinoids were also processed for comparative purposes. It was observed for the three analytes that the addition of the basic solution produced a rapid change in the color of the reaction mixture; after a few minutes, the color intensity reached a maximum. This was confirmed by registering the spectra of the reaction solutions over time. Representative results are shown in Figure 2. As observed in this figure, CBD formed an orange derivative with a maximum absorbance at 460 nm. CBN formed a pale reddish derivative with a maximum around 490 nm although the absorbances measured were much lower than those registered for CBD. Finally, THC led to the formation of a reddish solution, but the spectra did not show a maximum in the working wavelength range; instead, the spectra showed a shoulder around 492 nm. These values are consistent with those found in the literature [13,14,20]. The above wavelengths did not match with the wavelength of the maxima registered for the cannabis extracts (Figure 1A), which can be most probably explained by the fact that the extracts contained other cannabinoids.

In order to study the utility of the proposed reaction for quantitative purposes, the variation of the absorbances with the concentration of the analytes was tested. In this study, THC and CBD were used as model compounds. Solutions of the analytes at variable concentrations were processed, and the spectra were registered using a reaction time of 1 min. The final concentrations of analytes in the reaction solutions ranged from 10 to 50 ppm. The absorbances at 460 and at 492 nm for CBD and THC, respectively, were plotted against the concentration of analyte in the final mixture. The absorbances of a blank at such wavelengths were subtracted from the signal of the two analytes. Linear responses were observed for the two compounds. The corresponding calibration equations were y = (0.0109 ± 0.0005)x – (0.15 ± 0.02) for THC (R^2^ = 0.994, *n* = 6) and y = (0.0070 ± 0.0.0006)x + (0.01 ± 0.02) for CBD (R^2^ = 0.990, *n* = 5), respectively. The absorbances observed for CBN at the same concentrations were much lower, although the linear responses were observed for this compound at concentrations higher than 30 ppm. 

Figure 2 shows that the three tested cannabinoids originated with FBB different derivatives. Thus, the contribution of the derivative originated by each cannabinoid to the absorbance measured at a given wavelength is expected to be different. For example, at the working wavelength selected for the analysis of plant extracts (500 nm), the molar extinction coefficients measured for THC, CBD, and CBN were 3319, 1611, and 554 L mol^−1^ cm^−1^, respectively. Nevertheless, the absorbance values measured at such a wavelength correlate with the total concentration of the three cannabinoids in the working solution. According to the literature, THC, CBD, and CBN are the most abundant cannabinoids in in cannabis sativa materials [28]. Therefore, it can be assumed that the absorbance measured at 500 nm can be used for the rough estimation of the total content of cannabinoids in the working solutions (and thus, in the samples).

Next, the relationship between the amount of cannabinoids from the plant extracts in the reaction solution and the absorbances was studied. To this purpose, volumes of the extract obtained for a sample ranging from 6 to 100 µL were placed into the cuvettes, and water was added up to a final volume of 200 µL. Next, the 200 µL of the FBB reagent and 200 µL of the NaOH solutions were added. It has to be noted that the dilution factor of the extracts in the reaction mixture varied from 1:100 to 1:6 (*v/v*), equivalent to the volumes of extract per mL of the reaction mixture ranging from 10 µL to 166.67 µL. In Figure 1B are depicted the absorbances measured at 500 nm (after subtracting the signal of the blank) vs. the volume of sample extract per 1 mL of reaction mixture. As can be seen, the absorbances increased as the volume of the extract (and thus, the amount of cannabinoids) was increased. However, the increments were lower as the amount of the extract in the reaction mixture increased, which suggests the depletion of the FBB reagent. Although it was not tested, a better linearity could probably be achieved by using a more concentrated solution of reagent. 

The spectra of the sample extract in the absence of FBB were also recorded in order to evaluate their contribution to the final absorbances. Volumes of extracts ranging from 6 to 100 µL were diluted with water up to 400 µL, and then mixed with 200 µL of the NaOH solution. No significant absorbances in the 400–600 nm interval were observed for the resulting mixtures when the volume of extract used was ≤50 µL, equivalent to a dilution factor of the extract of 1:12 (Figure S3). This means that dilution factors ≥ than 1:12 are preferable. 

### 2.2. Reaction with FBB Immobilized into PDMS

#### 2.2.1. Study of the Reaction

For the reaction with the solid reagent immobilized into PDMS, an aliquot of the ethanolic extract (properly diluted with water) was mixed with the NaOH solution, and then the solid composite was immersed in the mixture (time zero of the reaction). In order to ensure a good contact between the composite and the working solutions, 400 µL of the diluted extracts and 400 µL of the NaOH solutions were used. 

**Figure 3:**
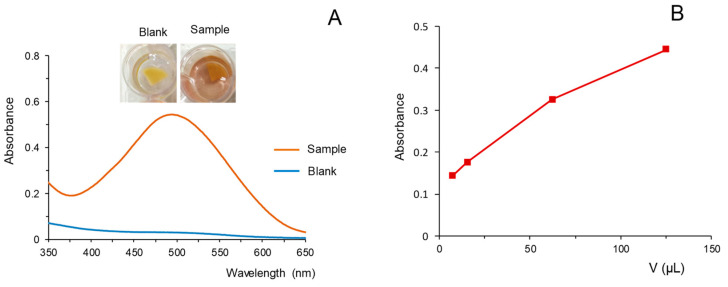
(**A**) Images and spectra obtained for a blank and an extract of cannabis using the reaction with FBB/PDMS. (**B**) Variation of the absorbance (corrected by the absorbance of a blank) as a function of the volume of sample extract per mL of reaction mixture. Reaction time, 1 min. For other experimental details, see text.

As illustrated in Figure 3A, in contact with the working solutions, it was observed that the FBB reagent diffused from the composites to the solutions producing a red-brown color similar to that observed when the extracts were treated with solutions of the reagent. 

**Figure 4:**
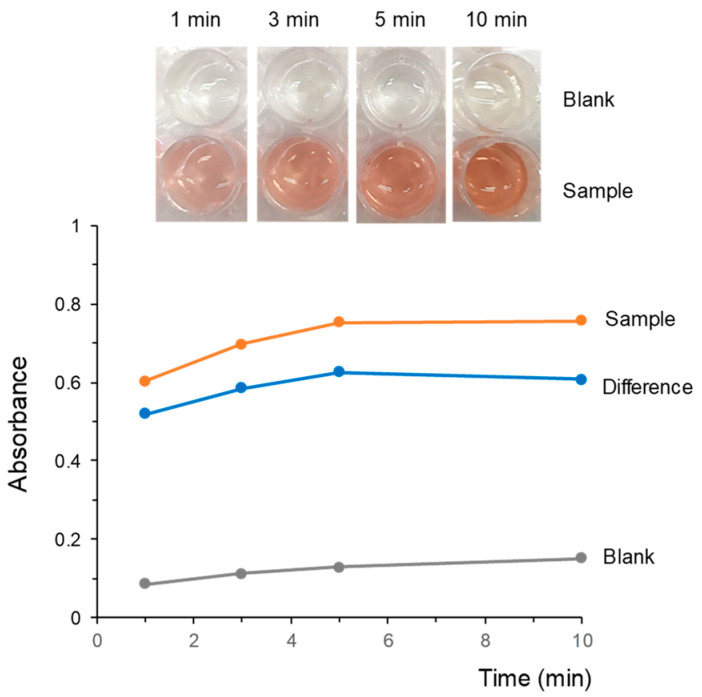
Images and variation of the absorbance at 500 nm with the time of exposure to the FBB/PDMS composites. Working solution, 50 µL of ethanolic extract of sample +350 µL of water +400 µL of 0.025 M NaOH. Time of exposure, 1 min. For other experimental details, see text.

Although the reaction was also fast and a positive response could be clearly observed in 1 min, the absorbances varied gradually during the first minutes of exposure to the FBB/PDMS composites. This is illustrated in Figure 4, which shows images of the solutions obtained for the same sample at different times of exposure. The absorbances at 500 nm of both a blank and the sample are also depicted in this figure. As observed, the absorbance of the blank also increased with time, indicating that FBB was gradually released from the composite to the working solution. The absorbance of the sample extracts shows a similar tendency, which confirms that, once released from the composites, FBB reacted very rapidly with the cannabinoids of the sample. As a result, when considering the differences of absorbances between the sample and the blank, little increment of the response with time was observed (see also Figure 4). For this reason, a time of exposure of 1 min was used for further experiments. It has to be remarked that, once the composites were removed from the working solutions, the absorbances remained constant, which confirmed that the derivatives originated were stable.

Next, assays with different volumes of the extract in the reaction solution were carried out. The volumes assayed ranged from 6.5 to 100 µL. The extracts were diluted with water up to 400 µL and mixed with 400 µL of the NaOH solution. In such a way, the volumes of extract assayed were equivalent to volumes of 8.125 µL to 125 µL per mL of reaction solution. The results obtained are depicted in Figure 3B. As observed in the figure, the variation of the absorbance was nearly linear within the tested range. These results indicate that, unlike the solution reaction (Figure 1B), the formation of product was not limited by the amount of reagent available in the working solution. This is consistent with the fact that the amount of solid FBB in the FBB/PDMS composites was much higher (≈680 µg) than the amount of FBB in the aliquot of reagent used in the solution reaction method (200 µL of a 25 µg/mL solution = 5 µg).

#### 2.2.2. Selectivity

**Figure 5:**
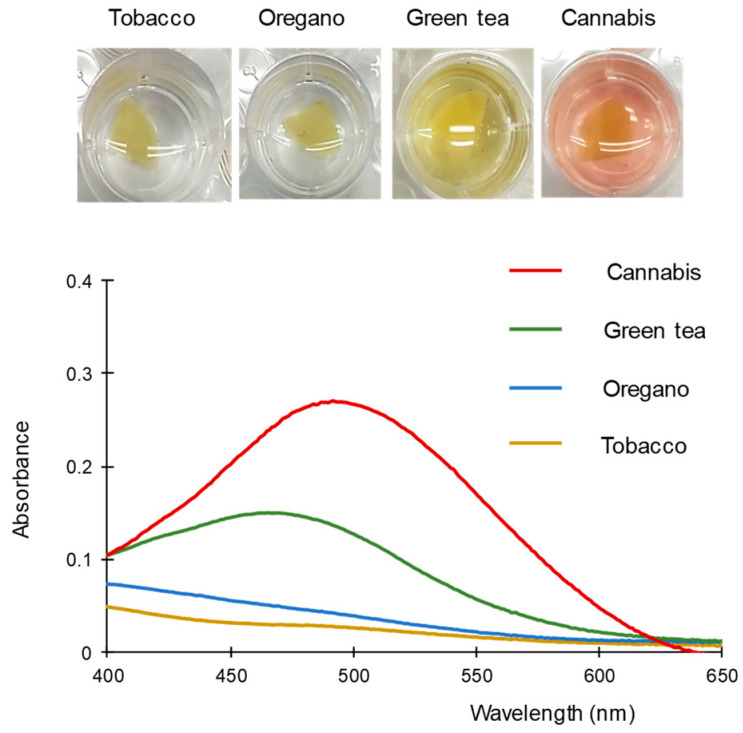
Images and spectra obtained with the proposed method for different plants. For other experimental details, see text.

To evaluate the selectivity, the proposed FBB/PDMS-based method was applied to the analysis of extracts obtained for other samples of botanical origin, namely tobacco, green tea, and oregano (dry samples). As shown in Figure 5, the extracts obtained from tobacco and oregano leaves did not produce a significant change in the color of the working solution with respect to that of a blank. The green tea sample led to a yellow solution, in part due to the extract itself. Nevertheless, the response could be clearly differentiated from a positive result for cannabinoids. 

It has been reported that FBB is also reactive towards other drugs [29]. For this reason, solutions of amphetamine, methamphetamine, cocaine, scopolamine, ephedrine, and pseudoephedrine were assayed. Potential excipients/adulterants such as procaine and caffeine were also tested. After 3 min of the reaction, a significant difference between the absorbances measured at 500 nm for the tested compounds and the blank was only found for methamphetamine (Figure S4), although the color of the resulting solution was yellow, and thus, clearly distinguishable from the color of samples positive for cannabinoids. 

#### 2.2.3. Application to Cannabis Samples

The proposed method was applied to analyze different cannabis samples. As stated earlier, the contribution of the extract to the total absorbance was negligible at 500 nm provided that the dilution factor of the extract in the working solutions was ≥1:12. For this reason, a volume of extract of 50 µL was used in quantitative studies. In such a way, the dilution factor of the extract was 1:16.

**Figure 6:**
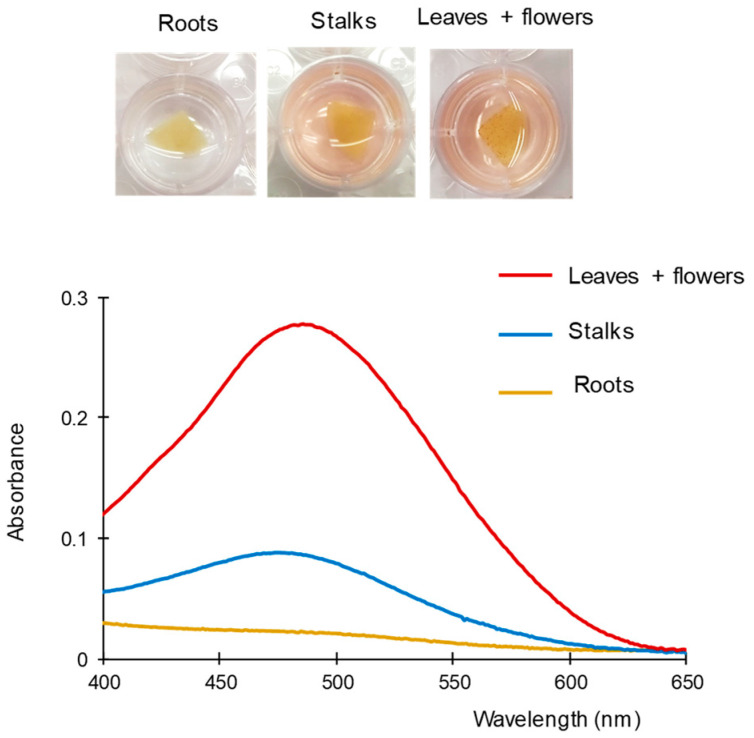
Images and spectra obtained with the proposed method for different parts of a cannabis plant. For other experimental details, see text.

First, different parts of a sample, namely roots, stalks, and leaves + flowers (buds), were manually separated from the bulk sample, and then homogenized. Portions of the three different parts were subjected to the proposed extraction conditions, and then treated with the FBB/PDMS composites. Next, the FBB/PDMS composites were introduced into the working solution for 1 min. Finally, the composites were removed and the absorbances of the resulting solutions were measured. The results obtained are shown in Figure 6. The amount of cannabinoids found in the roots was negligible. As expected, the highest level of cannabinoids was found in the samples composed of flowers and leaves. These results are similar to those reported by other authors using chromatographic techniques [18].

Different cannabis samples (leaves and flowers) were processed with the FBB/PDMS composites, and the content of cannabinoids was estimated through the measurement of the absorbances at 500 nm. The results were expressed as CBD. With this aim, a calibration equation was previously obtained by processing standard solutions of CBD by the FBB/PDMS approach. The concentrations assayed ranged from 10 to 50 ppm (values referred to the reaction solution). The equation obtained by using the absorbances at 500 nm corrected by the absorbance of a blank was y = (0.0325 ± 0.0017)x + (0.08 ± 0.04) (*n* = 7, R^2^ = 0.990). The limit of detection of the method (calculated as 3 x standard deviation of the intercept/slope of the calibration line) was 3.7 ppm. The reproducibility was estimated through the successive measurement of the concentration of total cannabinoids in three different extracts of samples (leaves and flowers). The results, expressed as the relative standard deviation, were ≤9%. Since no reference method was available, recovery studies with fortified samples were undertaken to study the accuracy [30]. For one of the samples (S1), portions of the extracts were fortified with known amounts of CBD, and the differences of absorbances measured for the unspiked and spiked extracts were transformed in concentration of CBD using the above equation. The concentration found in the spiked extract was (98 ± 3)% of the amount added. These results indicate that, under the proposed conditions, the accuracy of the method was not affected by the matrix. Additional recovery tests with the other samples used along the study were not carried out because all of them had similar matrices (forensic cannabis sativa). Finally, the proposed method was applied to measure the content of cannabinoids in different cannabis samples. The results obtained are summarized in Table 1.

As observed in Table 1, the values found ranged from 10.9% to 23.7%, with S4 and S1 being the samples with the lowest and highest content of cannabinoids, respectively. One of the samples was analysed using two different volumes of the extract, equivalent to dilution factors of 1:16 and 1:32. The results obtained were statistically comparable at a 95% confidence level (t_calculated_ = 2.06, t_theoretical_ = 4.303). The results of Table 1 also demonstrate that the proposed method provides satisfactory precision, as the relative standard deviations found were ≤9%. 

## 3. Discussion

For decades, Cannabis sativa L. and derived products have been the most widely consumed drugs around the world, as well as used for therapeutical reasons [31]. In addition, during the past years, an enormous interest in substances present in cannabis plants has arisen because of the potential benefits of some of their constituents, mainly CBD, in human health. This interest has generated a great expansion of the market for cannabis-related products. As a result, there is an increasing demand for chemical information about cannabis plants in the forensic, toxicological, and medical fields, as well as in nutraceutical, food, and cosmetics industries, among others [1,2]. In this respect, LC is currently the technique of choice, and several chromatographic assays can be found in the literature that allow the accurate determination of cannabinoids in a variety of samples [6]. The individual content of the main cannabinoids may be necessary for different purposes. For example, the percentage of THC is necessary to verify the legality of the samples. The amount of CBD is commonly used for evaluating the quality of the plant materials intended for the preparation of products with therapeutical applications, whereas the content of CBN can be used to assess the preservation of the samples [4]. The sum of the percentages obtained for the individual cannabinoids through a chromatographic analysis can be used to estimate the potency of samples [18,32]. 

The method developed in the present work can be considered a complementary tool to such well-established methodologies for the rough estimation of the total content of cannabinoids. The assay could be used to control variations in the percentage of cannabinoids during the production of the plants, to compare plants of similar characteristics, or to check the quality of raw materials and residues generated during the industrial processes involving cannabis material [5,19,28]. The method could be adapted to estimate the total content of cannabinoids in other types of cannabinoid-containing materials such as foods or cosmetic products, after applying a proper sample treatment according to the characteristics of the matrix [7]. Although the method was intended for the rapid estimation of the total content of cannabinoids, it could be also used in combination with chemometric tools to obtain further information on the content of the individual cannabinoids, taking advantage of the spectral differences between their respective FBB derivatives.

The main advantages of the proposed approach are simplicity and speed, as the information can be obtained with a minimum of instrumentation (a balance and a colorimeter). A simple ultrasonication is required for sample preparation, although the method can be further simplified by replacing ultrasonication by manual stirring (see Figure S2). Moreover, the solvent used for extraction is not harmful. As regards the reaction, the employment of solid FBB immobilized in PDMS results in a very simple procedure (only a solution of NaOH is needed). It has to be remarked that in the course of our study, stable responses were obtained with composites kept at 4 °C and in the dark for at least 10 days. The selectivity found when testing other samples of botanical origin and common drugs was satisfactory.

As stated earlier, no reference methods are available for the estimation of the total content of cannabinoids. Moreover, the content of cannabinoids in cannabis is highly variable. Although not strictly comparable, the results obtained with the proposed method can be considered compatible with values found in the literature considering the sum of the percentages reported for the individual major cannabinoids [3,4,8,32,33,34]. 

## 4. Materials and Methods

### 4.1. Reagents and Solutions

All the reagents were of analytical grade. Standard solutions in methanol of Δ9-*trans*-tetrahidrocannabinol (THC), cannabidiol (CBD), and cannabinol (CBN) were obtained from Sigma-Aldrich (St. Louis, MO, USA). Ethanol and methanol were purchased from VWR (Radnor, PA, USA). Sodium hydroxide and Fast Blue B salt (FBB) were obtained from Sigma-Aldrich. The PDMS kit (Sylgard^®^ 184 silicone elastomer base and Sylgard^®^ 184 silicone elastomer curing agent) was obtained from Dow Corning (Midland, MI, USA).

Intermediate stock standard solutions of the three cannabinoids tested (1000 µg/mL) were prepared by diluting the commercial standards with acetonitrile and kept at −20 °C until use. Working solutions were prepared by diluting the stock solutions with ultrapure water. Solutions of FBB at a concentration of 25 µg/mL and 0.025 M NaOH were prepared by dissolving the appropriate amounts of the solid reagents in water.

Ultrapure water was obtained from an Adrona system (Riga, Latvia).

### 4.2. Instrumentation and Conditions

For the spectrophotometric measurements, a UV-vis Agilent 8453 diode-array UV spectrophotometer (Agilent Technologies, Waldbronn, Germany) was used. The spectra were recorded in the 350–650 nm range. An ultrasonic bath (300 W, 40 kHz, Sonitech, Guarnizo, Spain) was used for sample treatment. 

### 4.3. Preparation of the FBB/PDMS Composites

For the preparation of the composites, the FBB solid reagent was first ground in a glass mortar until a fine powder was obtained, and then sieved (<120 µm). Next, 30 mg of the reagent was mixed with 2 g of PDMS elastomer base. The mixture was stirred vigorously. Next, 0.2 g of the curing agent was added, and the resulting mixture stirred until homogeneity. Portions of 0.2 g of the resulting mixtures were placed in circular well polystyrene plates of 15 mm diameter. The mixtures were cured at room temperature in the dark for 3 days. The solid composites were removed from the well plates and cut into four pieces of approximately the same size and stored at 4 °C in the dark until use.

### 4.4. Reaction Conditions

For the reaction in solution studies, aliquots of 200 µL of the standard solutions or the extracts (or diluted extracts) of the samples were introduced in the cuvettes and mixed with 200 µL of a solution of FBB (25 µg/mL). Next, 200 µL of a solution of 0.025 M NaOH was added, and the absorbance spectra of the resulting solutions were registered after a given reaction time (1 min in the optimized procedure) within the 350–650 nm range.

For the reaction with the composites, aliquots of 400 µL of the standard solutions or of the extracts (previously diluted with water) were placed in wells of a well plate and mixed with 400 µL of 0.025 M NaOH. Then, the composites (quarters) were introduced into the mixture. After a time of exposure (1 min in the optimized procedure), the resulting solutions were transferred to a cuvette, and their spectra were recorded in the 350–650 nm range.

### 4.5. Analysis of the Cannabis Samples

Different recreational cannabis samples, donated voluntarily by individuals who were previously informed of the aim of the study, were used throughout the study. Portions of the homogenized samples were suspended in ethanol (unless otherwise stated), and then treated in an ultrasonic bath for 10 min. The liquid phase was then separated and kept in the dark at 4 °C until analysis. Before analysis, the extracts were properly diluted with water in order to adjust the absorbance values of the product of reaction with FBB to the desired values. 

## 5. Conclusions

In the present work, a convenient option for the rough estimation of the total content of cannabinoids in cannabis sativa has been developed, using the colorimetric reagent FBB immobilized into PDMS. The employment of the FBB-PDMS composites offers some advantages over the reaction solution, mainly an extended linear working interval for quantitative applications, eliminating the requirement of preparing a solution of the reagent in the moment of the analysis. The results obtained can be referred to any of the individual cannabinoids; in the present instance, we have selected CBD to express the total percentage of cannabinoids. As an example of application, the proposed method has been applied to compare the content of cannabinoids in different samples intended for recreational usage. 

## Figures and Tables

**Table 1:** Results obtained in the analysis of different cannabis samples with proposed procedure (*n* = 3).

Sample	Volume of Ethanolic Extract in the Reaction Solution ^1^ (µL)	Content of Cannabinoids, Expressed as CBD (%)
S1	25	22.5 ± 0.13
S1	50	23.7 ± 1.0
S2	50	16.8 ± 1.3
S3	50	14.2 ± 1.3
S4	50	10.9 ± 0.6
S5	50	18.6 ± 0.8

^1^ final volume of the reaction solution, 800 µL.

## Data Availability

Data are contained within the article or supplementary material.

## Supplementary Materials

The following supporting information can be downloaded at: https://www.mdpi.com/article/10.3390/molecules28031303/s1, Figure S1. Variation of the absorbance at 500 nm with the time of reaction in the solution reaction approach for a sample (25 µL of methanolic extract of a cannabis plant sample + 175 µL of water) and for a blank (200 µL of water). For other experimental details, see text. Figure S2. Spectra obtained for cannabinoids present in a cannabis sample: (A) extracts obtained with methanol and ethanol; (B) extracts obtained with two successive extractions with; (C) extracts obtained with ethanol by using ultrasonication and hand stirring for 10 min.; Figure S3. Spectra obtained in absence of FBB for different volumes of sample extract. Reaction media, sample extract + water up to 200 µL and 200 µL of 0.025 M NaOH. For other experimental details, see text; Figure S4. Spectra obtained for different drugs derivatized with FBB under the solution reaction conditions.

## Sample Availability

Samples of the compounds are not available from the authors.
