# Supplementary material for: A Colorimetric Method for the Rapid Estimation of the Total Cannabinoid Content in Cannabis Samples
Source: Molecules. 2023 Jan 30;28(3):1303. doi: 10.3390/molecules28031303 (PMC9921926; doi:10.3390/molecules28031303)
Supplement: Supplementary file 1 [file molecules-28-01303-s001.zip › molecules-2117971-supplementary.pdf]

Supplementary content

A colorimetric method for the rapid estimation of the total cannabinoids content in cannabis samples

Neus Jornet-Martínez[1], Josep Biosca-Micó, Pilar Campíns-Falcó[1], Rosa Herráez-Hernández[1]

[1] MINTOTA Research Group, Departament de Química Analítica, Facultat de Química, Universitat de València, Dr. Moliner 50, 46100-Burjassot, Spain

* Correspondence: rosa.herraez@uv.es (R. H.-H.); Tel.: +34-96-3544978

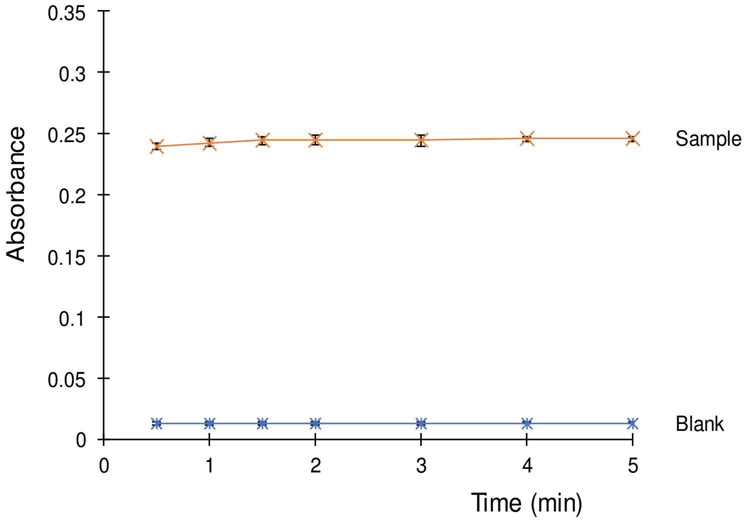

Figure S1. Variation of the absorbance at 500 nm with the time of reaction in the solution reaction approach for a sample (25 µL of methanolic extract of a cannabis plant sample + 175 µL of water) and for a blank (200 µL of water). For other experimental details, see text.

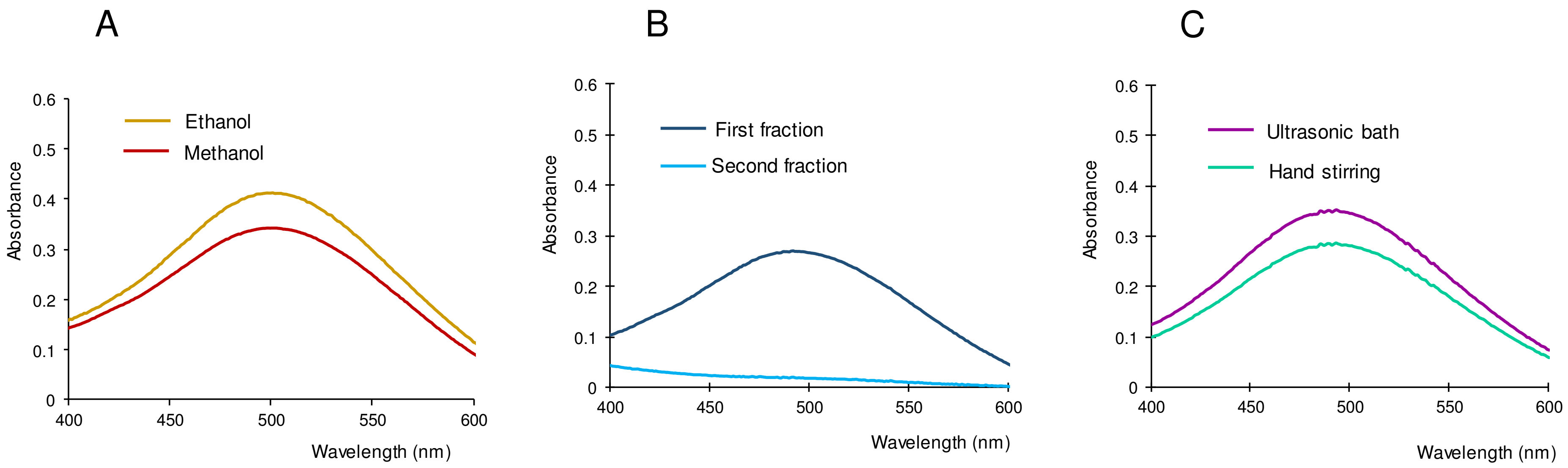

Figure S2. Spectra obtained for cannabinoids present in a cannabis sample: (A) extracts obtained with methanol and ethanol; (B) extracts obtained with two successive extractions with; (C) extracts obtained with ethanol by using ultrasonication and hand stirring for 10 min. For other experimental details, see text.

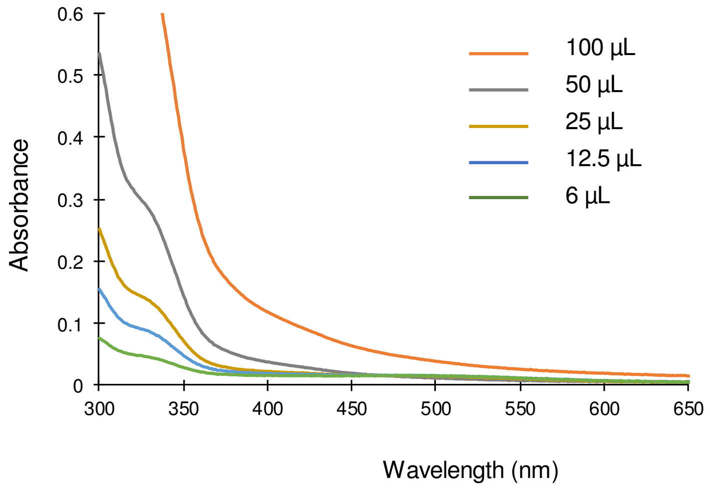

Figure S3. Spectra obtained in absence of FBB for different volumes of sample extract. Reaction media, sample extract + water up to 200 µL and 200 µL of 0.025 M NaOH. For other experimental details, see text.

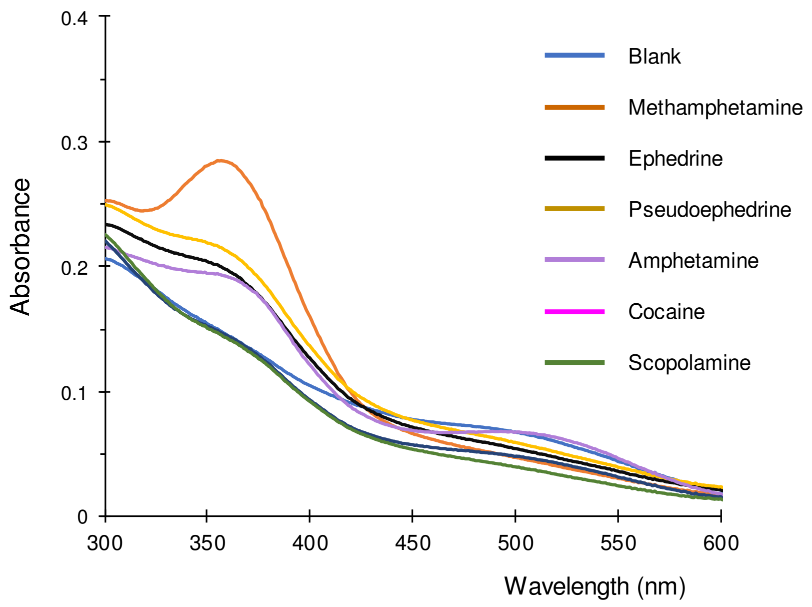

Figure S4. Spectra obtained for different drugs derivatized with FBB under the solution reaction conditions.
